# Molecular Characterization and Antimicrobial Susceptibility of *Streptococcus pneumoniae* Isolated from Children Hospitalized with Respiratory Infections in Suzhou, China

**DOI:** 10.1371/journal.pone.0093752

**Published:** 2014-04-07

**Authors:** Qian Geng, Tao Zhang, Yunfang Ding, Yunzhen Tao, Yuzun Lin, Yunzhong Wang, Steven Black, Genming Zhao

**Affiliations:** 1 Department of Epidemiology, School of Public Health, Fudan University, Key Laboratory of Public Health Safety, Ministry of Education, Shanghai, China; 2 Soochow University Affiliated Children's Hospital, Suzhou, China; 3 Center for Global Health, Cincinnati Children's Hospital, Cincinnati, Ohio, United States of America; St. Petersburg Pasteur Institute, Russian Federation

## Abstract

**Background:**

Dissemination of antibiotic resistant clones is recognized as an important factor in the emergence and prevalence of resistance in pneumococcus. This study was undertaken to survey the antimicrobial susceptibility and serotypes distribution of pneumococci and to explore the circulating clones in hospitalized children in Suzhou, China.

**Methods:**

The pneumococci were isolated from the nasopharyngeal aspirates of children less than 5 years of age admitted to Soochow-University-Affiliated-Children's-Hospital with respiratory infections. The capsular serotypes were identified by multiplex polymerase chain reaction (PCR). Antimicrobial susceptibility was tested by E-test. The presence of *ermB*, *mefA/E* genes were detected by PCR and the genotypes were explored by Multilocus sequence typing (MLST).

**Results:**

From July 2012 to July 2013, a total of 175 pneumococcal isolates were collected and all strains were resistant to erythromycin and clindamycin, about 39.4% strains were non-susceptible to penicillin G. Overall, 174 (99.4%) isolates were resistant to ≥3 types of antibiotics. Serotypes 19F (28.1%), 6B (19.7%), 19A (18.0%), and 23F (17.4%) were the most common serotypes in all identified strains. The serotypes coverage of PCV7 and PCV13 were 71.9% and 89.9%, respectively. Four international antibiotic-resistant clones, including Taiwan^19F^-14 (n = 79), Spain^23F^-1(n = 25), Taiwan^23F^-15(n = 7) and Spain^6B^-2(n = 7), were identified. The Taiwan^19F^-14 clones have a higher non-susceptibility rate in β-lactams than other clones and non-clone isolates (p<0.001). In addition, 98.7% Taiwan^19F^-14 clones were positive of both *ermB* and *mefA/E* genes, compare to 33.3% in other clones and non-clone strains.

**Conclusions:**

The spread of international antibiotic-resistant clones, especially Taiwan^19F^-14 clones, played a predominant role in the dissemination of antimicrobial resistant isolates in Suzhou, China. Considering the high prevalence of PCV7 serotypes and serotype 19A, the introduction of PCV13 may be a promising preventive strategy to control the increasing trend of clonal spread in China.

## Introduction

*Streptococcus pneumoniae* (*S.pneumoniae*), an important bacterial pathogen responsible for respiratory infections, remains a leading cause of morbidity and mortality in infants and younger children. In 2005, the World Health Organization (WHO) estimated that 0.7–1 million children, mostly from developing countries, died of pneumococcal disease annually [1]. The increasing trend of *S.pneumoniae* antimicrobial-resistance and emergence of multidrug- resistant (MDR)*S.pneumoniae* isolates, which may result from inappropriate use of antibiotics, has been a worldwide concern [2], [3], [4], [5]. The situation has been especially problematic in many Asian countries, China in particular. A surveillance study from the Asian Network for Surveillance of Resistant Pathogens (ANSORP) showed that the rate of erythromycin resistance (96.4%) and MDR (83.3%) in China ranked the highest among 11 Asian countries [6], which underlines the urgent need for preventive strategy to control pneumococcal disease in China.

The heptavalent pneumococcal conjugate vaccine (PCV7) has a dramatic effect in reducing the disease burden of pneumococcal diseases, especially in decreasing the incidence of invasive pneumococcal diseases (IPD) and pneumonia [7], [8], [9], [10]. However, the remarkable increased incidence of pneumococcal diseases caused by non-vaccine serotypes, especially 19A, has been repeatly documented in the post PCV7 era [11], [12], [13]. In mainland China, PCV7 was introduced in September 2008 for optional use, but the vaccine has not been widely used in most parts of China. Previous studies have shown that PCV7 serotypes coverage varied across China, and a trend of serotypes changing have been reported recently [6], [14], [15].

Pneumococcal epidemiology and seroepidemiology make it complex with the organism responding to environmental pressures such as antibiotic use and vaccine introduction. Pneumococci are capable of undergoing capsular switching and the current conjugate vaccine will likely result in an increase in the amount of pneumococcal infections caused by non-vaccine serotypes. Thus only focusing on the changing trend of serotypes and antimicrobial susceptibility of pneumococci is not enough to monitor the epidemiology and clonal spread of the pneumococci [16]. Many studies have revealed that the worldwide pneumococcal diseases are largely caused by a few multidrug-resistant clones [17], [18]. However, lineages have diversified natural transformation and genome recombination of *S. pneumoniae* in response to clinical interventions [19].

Therefore, through describing the antibiotic resistance patterns, serotype distribution and molecular characteristics of *S.pneumoniae* carried by hospitalized children with respiratory infections from Suzhou, China, we explored the prevalent clones and evolution of pneumococcal population.

## Materials and Methods

### Study site and study population

This study was conducted from July 2012 to July 2013 at Soochow University Affiliated Children's Hospital (SCH), the sole tertiary children's hospital in Suzhou district. Routinely, nasopharyngeal aspirates were collected for all children admitted to SCH due to respiratory infections to detect carriage of pathogens in the first morning after admission. Nasopharyngeal aspirates collected every Sunday and Monday that were positive for *S. pneumoniae* (screening at Tuesday and Wednesday) in children younger than 5 years of age, were included in the study.

### Bacterial isolates

The nasopharyngeal aspirates obtained were transported to the microbiology laboratory of SCH in sterile saline within 2 h. The specimens were cultured on agar plates supplemented with 5% defibrinated sheep's blood and incubated overnight at 37°C in 5% CO_2_ atmosphere. *S.pneumoniae* was identified and confirmed by typical colony morphology, alpha-hemolysis, Gram staining, Optochin (Oxoid, Basingstoke, UK) susceptibility and bile solubility. All strains were stored at −80°C on porous beads.

### Antimicrobial susceptibility test

The minimal inhibiting concentrations (MICs) of antimicrobial agents, including erythromycin, penicillinG, co-trimoxazole, vancomycin, cefotaxime, ceftriaxone clindamycin, tetracycline, amoxicillin, chloromycetin and levofloxacin were measured using the E-test methodology (AB BioDisk, Switzerland). *S.pneumoniae* ATCC49619 was used as a quality control strain in antimicrobial susceptibility tests. The interpretations of MIC breakpoints and test results were made according to the recommended method of the Clinical and Laboratory Standards Institute (CLSI)criteria [20]. Isolates not susceptible to at least three antibiotic families were defined as multidrug-resistant (MDR) *S.pneumoniae*.

### DNA extraction

Chromosomal DNA was extracted from subculture of *S.pneumoniae* isolates by lysozyme and silicon substrate column adsorption method using TIANamp Bacteria DNA Kit (TIANGEN BIOTECH, Beijing, China) according to the manufacturer's instructions.

### Serotyping

The serotypes of pneumococcal isolates were identified by a multiplex polymerase chain reaction (PCR) method as described in previous studies [21], [22], [23]. Nineteen different serotypes were determined by 5 sequential multiplex PCR reactions: reaction 1 includes serotypes 6A/B, 9V, 15B/C, 18C, 19F, reaction 2 includes serotypes 3, 14, 19A, 23F, reaction 3 includes1, 4, 5, 23A, reaction 4 includes15A, 7F, 22F and reaction 5 includes serotype 20, 34, 33F. The serotypes not included in the multiplex PCR reactions were defined as non-typed serotypes.

### Detection of macrolide resistant genes

The macrolide resistant genes *ermB* and *mefA/E* were amplified by PCR methods for all erythromycin-resistant isolates [24]. A positive reference control strain was included in all PCR experiments. The PCR products were run on a 2% agarose gel electrophoresis and were visualised by ethidium bromide staining.

### Multilocus sequence typing (MLST)

Sequence types (ST) of *S.peunomiae* isolates were determined using multilocus sequence typing(MLST) technique. The internal fragments of 7 housekeeping genes *(aroE, gdh, gki, recP, spi, xpt, ddl*) were amplified from chromosomal DNA by PCR methods [25]. The STs were determined by the comparison with those of corresponding allelic profiles at MLSTdatabase (http://spneumoniae.mlst.net). The new STs and alleles were submitted to the curator of MLST website for assignments. eBURSTV3 software (http://eburst.mlst.net) was used to explore the relationships among isolates and to group the STs sharing 6 identical alleles of seven loci into a clonal complex (CC).

### Statistical analysis

All the statistical analyses were performed by using the SPSS statistical package version 16.0 (SPSS, Chicago, Illinois, USA). The Chi-square test was performed for comparing the proportions. All tests were two-tailed and P values less than 0.05 were considered statistically significant.

### Ethics statement

The study was approved by the Institute Review Board (IRB) of School of Public Health, Fudan University. Written informed consent was obtained from parents or guardians on behalf of children participants involved in the study before enrollment.

## Results

### The demographic and clinical characteristics of enrolled subjects

A total of 175 children out of 183 enrolled children with nasopharyngeal aspirates positive for *S. pneumoniae* were finally included. The median age for patients was 12.7 months or 1.1 years(IQR: 7.9–23.8 months or 0.66–1.98 years). About 89.7% (n = 157) of patients were diagnosed with pneumonia. Pneumonia would be diagnosed when the children had at least 2 of the following clinical manifestations: cough, respiratory secretions, abnormal auscultation, dyspnea; and/or Chest X-ray infiltrates. One hundred twenty one (69.1%) cases were boys and 132 (75.5%) were younger than 2 years old (Table 1).

**Table 1:** The demographic and clinical characteristics of 175 enrolled patients.

Characteristics		No.	Percentage (%)
Gender	Male	121	69.1
	Female	54	30.9
Age (y)	0-	82	46.9
	1-	50	28.6
	2-	14	8.0
	3-	22	12.6
	4–5	7	4.0
Diseases	Pneumonia	157	89.7
	Upper respiratory infections	4	2.2
	Septicemia	2	1.1
	Others^a^	12	6.9

Note:

a Others included bronchitis, asthma, acute bronchiolitis and acute laryngotracheal bronchitis.

### Antimicrobial susceptibility

All the 175 *S.pneumoniae* isolates were resistant to erythromycin and clindamycin, but susceptible to vancomycin and levofloxacin. The overall tetracycline and co-trimoxazole non-susceptible rates were as high at 94.9% and 94.3%, respectively, while the resistant rate to chloromycetin was merely 4.6%. In terms of β-lactams antibiotics, the non-susceptible rates to ceftriaxone, penicillin G, amoxicillin and cefotaxime were 38.7%, 39.4%, 42.3% and 55.4%, respectively (Table 2). The non-susceptible rate of *S. pneumoniae* to penicillin G isolated from children younger than 2 years was lower than that of 2–5 years children (34.8% vs. 53.5%, χ^2^ = 4.72, P = 0.03)

**Table 2:** Antimicrobial susceptibility and MICs of 175 *S.pneumoniae* strains isolated from respiratory infection Children younger than 5 years.

Antibiotic agents	MIC (μg/ml)			No. of isolates (%)			Non-susceptible rate (%)
	50%	90%	range	Resistant	Intermediate	Susceptible	
Erythromycin	≥1	≥1	≥1	175(100.0)	0(0.0)	0(0.0)	100.0
Penicillin G	2	≥8	≤0.031–≥8	20(11.4)	49(28.0)	106(60.6)	39.4
Cefotaxime	2	≥4	≤0.06–>4	43(24.6)	54(30.9)	78(44.6)	55.4
Ceftriaxone	1	≥4	≤0.06–≥4	14(22.6)	10(16.1)	38(61.3)	38.7
Amoxicillin	≤2	≥8	≤0.06–≥8	44(25.1)	30(17.1)	101(57.7)	42.3
Co-trimoxazole	≥4	16	≤0.5–≥32	147(84.0)	19(10.9)	9(5.1)	94.9
Clindamycin	≥1	≥1	≥1	175(100.0)	0(0.0)	0(0.0)	100.0
Tetracycline	≥8	≥16	≤1–≥16	163(93.2)	2(1.1)	10(5.7)	94.3
Levofloxacin	≤2	≤2	≤0.5–≤2	0(0.0)	0(0.0)	175(100.0)	0.0
Chloromycetin	≤4	4	≤2–≥32	8(4.6)	0(0.0)	167(95.4)	4.6
Vancomycin	≤1	≤1	≤1	0(0.0)	0(0.0)	175(100.0)	0.0

Except for one isolate (0.6%) that was only resistant to erythromycin and clindamycin, all other 174 (99.4%) isolates were MDR strains and non-susceptible to 3 or more antibiotics. The most common MDR patterns were erythromycin/co-trimoxazole/clindamycin/tetracycline/β-lactams (58.9%), followed by erythromycin/co-trimoxazole/clindamycin/tetracycline (26.9%).

### Serotype distribution and vaccines coverage

**Figure 1:**
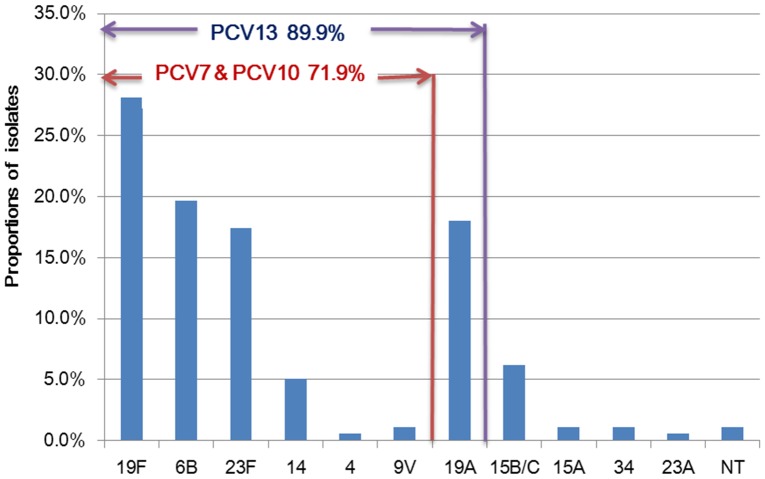
The serotype distribution of the *S.pneumoniae* isolates. Note: Given to 3 co-colonization isolates, the denominator of PCV coverage rate and serotype proportions was 178. NT indicated non-typed serotypes.

Except for 2 (1.1%) non-typed isolates, all isolates were typed into 11 serotypes and 3 co-colonized isolates with two serotypes (19A and 6B). The most prevalent serotypes were 19F (n = 50, 28.1%), 6B (n = 35, 19.7%), 19A (n = 32, 18.0%) and 23F (n = 31, 17.4%), accounting for 83.2% of the isolates. There were 128 (71.9%) isolates included in PCV7 and PCV10 serotypes, as well as 160 (89.9%) isolates grouped into PCV13 serotypes (Figure 1). No significant difference was detected in serotype distribution between the two age groups children: younger than 2 years and 2–5 years (P>0.05).

### Molecular typing

Except for the failure of sequencing in one serotype 6B strain's *spi* allele, all other 174 *S.pneumoniae* isolates were successfully typed by MLST. Among the 43 sequence types (STs) identified, 17 STs were newly assigned (ST8908-8916, ST9062-9063, ST9110-9114) and 76.5%(13/17) of new STs were novel combination of known alleles. The single new allele was *spi*389. The most common STs were ST271 (n = 36, 20.6%), ST320 (n = 32, 18.3%), ST81 (n = 22, 12.6%) and ST3173 (n = 14, 8.0%). Most of ST320 isolates were serotyped as 19A alone (except for 3 ST320 isolates were 19A and 6B), and all ST271, ST81 and ST3173 were typed into serotype 19F, 23F and 6B, respectively.

**Figure 2:**
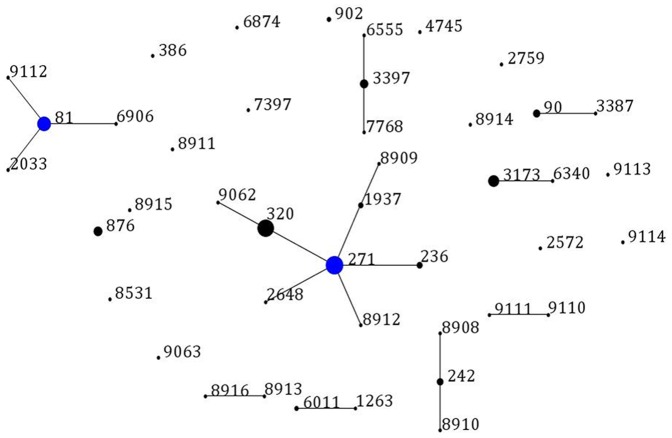
Population snapshot of 174 *S.pneumoniae* isolates. One spot represents a single ST. The size of the circle corresponds to the number of isolates belonging to a ST. The lines indicate the presence of single locus variant (SLV) links among particular STs. Blue spots indicate the founder of a clonal complex.

The eBURSTv3 analysis revealed 9 clonal complexes (CCs) and 15 singletons containing 150 and 24 isolates, respectively (Figure 2). The predominant international antibiotic-resistant CCs were CC271 (Taiwan^19F^-14 clones, n = 79, 45.4%), CC81 (Spain^23F^-1 clones, n = 25, 14.4%), CC242 (Taiwan^23F^-15 clones, n = 7, 4.0%) and CC90 (Spain^6B^-2 clones, n = 7, 4.0%). Overall, 67.4% of isolates were grouped to the international antibiotic-resistant clones.

In further analysis, we found that the rate of non-susceptibility to penicillin G of all international antibiotic-resistant clones was 50.0%, which is significantly higher than that of other non-clone isolates(χ^2^ = 16.40, P<0.001). The non-susceptibility to cefotaxime (χ^2^ = 15.07,P<0.001) and amoxicillin (χ^2^ = 23.65,P<0.001) between the clones and non-clone strains were significantly different. Moreover, Taiwan^19F^-14 clones had the highest non-susceptible rate to β-lactams antibiotics among all the clone strains identified in this study(χ^2^_penicillin G_ = 11.07, P_penicillin G_  = 0.001;χ^2^_cefotaxime_ = 7.03, P_cefotaxime_ = 0.008; χ^2^_amoxicillin_ = 42.06, P_ amoxicillin_<0.001) (Table 3).

**Table 3:** The antimicrobial susceptibility and MIC s of β-lactams antibiotics between international antibiotic-resistant clones and other strains.

Clones^a^	No.	Penicillin G		Cefotaxime		Amoxicillin	
		MIC50/90 (μg/ml)	No. of non-S (%)	MIC50/90 (μg/ml)	No. of non-S (%)	MIC50/90 (μg/ml)	No. of non-S (%)
Taiwan^19F^-14	79	4/≥8	48(60.8)	2/>4	58(73.4)	4/≥8	60(75.9)
Spain^23F^-1	25	2/4	6(24.0)	1/2	11(44.0)	≤2/4	2(8.0)
Taiwan^23F^-15	7	2/≥8	2(28.6)	2/2	4(57.1)	≤2/4	1(14.3)
Spain^6B^-2	7	2/4	3(42.9)	2/4	4(57.1)	≤2/4	2(28.6)
Clones	118	4/≥8	59(50.0)	2/>4	77(65.3)	4/≥8	65(55.1)
Other isolates	56	2/4	10(17.9)	1/4	19(33.9)	≤2/4	9(16.1)

Note:

a Clones represented the four international antibiotic-resistant clones including Taiwan^19F^-14, Spain^23F^-1, Taiwan^23F^-15 and Spain^6B^-2.

### Presence of macrolide-resistant genes

There were 62.9% of (110/175) isolates positive for both *ermB* and *mefA/E* genes, and 37.1% (65/175) positive for *ermB* gene alone (Table 4). Overall 98.7% of Taiwan^19F^-14 clones harbored both *ermB* and *mefA/E* genes, whereas 33.3%(32/96) of other clones and non-clone isolates harbored *ermB* and *mefA/E* genes (χ^2^ = 79.40, P<0.001).

**Table 4:** The distribution of macrolide-resistant genes among 175 *S.pneumoniae* isolates in different clones.

Macrolide-resistant genes		No. (%)	Clones				
*ermB*	*mefA/E*		Taiwan^19F^-14	Spain^23F^-1	Taiwan^23F^-15	Spain^6B^-2	Other isolates a
+	+	110(62.9)	78(98.7)	8(32.0)	3(42.9)	1(14.3)	20(35.1)
+	-	65(37.1)	1(1.3)	17(68.0)	4(57.1)	6(85.7)	37(64.9)

Note:

a The one isolates without ST number was grouped into other isolates.

## Discussion

Given that the blood culture isolates are rarely obtained from children with pneumonia and induced sputa in children are difficult to obtain, we relied upon nasopharyngeal aspirates to assess potential pathogens in the children in our study. These isolates could represent colonization or the causative agents of these infections. Pneumococcus infections in children are likely due to colonizers that may be the reistance strains. Thus, we expect that the results of our study are representative of the likely contribution of resistant strains in children in our population.

In the present study, the non-susceptible rate of pneumococci to penicillin was 39.4% and increased with age. This situation may result from the widespread use of penicillin in older children. Using the same CLSI criteria, the non-susceptible rate to penicillin in our study was higher than that in Beijing(0.7%) [26]. The non-susceptible rate to ceftriaxone in our study was higher than that of other non-invasive pneumococcal strains(4.6%) as well [6]. In addition, 99.4% of pneumococcal isolates were resistant to mutiple antibiotics, showing a much higher resistant rate than that 83.3% in china and 59.4% in other Asian countries [6]. These differences of antimicrobial susceptibility may be explained by the differences of the source population and antibiotics usage. All the pneumococcal strains in our study were isolated from children and most of them had antibiotics before sampled. While the study population mentioned above were all age groups and the antibiotics usage was much less in adult population.

Pneumococcal macrolide resistance was mediated by two major mechanisms: target modification by a ribosomal methylase encoded by the *ermB* gene and drug efflux encoded by the *mefA/E* gene [27], [28]. The *ermB* gene was generally associated with high level of macrolide resistance (MIC>64 μg/ml), while the *mefA/E* gene was associated with low level of macrolide resistance (MIC:1–32 μg/ml) [27], [28]. In the present study, all of the 175(100.0%) pneumococcal isolates were resistant to erythromycin, 62.9% of pneumococcal isolates expressed both *ermB* and *mefA/E* genes, and 37.1% isolates expressed *ermB* gene only. The dramatically high proportion of isolates with both *ermB* and *mefA/E* genes was associated with the remarkably high MDR rate in Suzhou.

The predominant *S.pneumoniae* serotypes in our study were 19F, 6B, 19A, 23F, accounting for 83.2% of the isolates. Compared to the strains isolated in Suzhou during 2006–2007, the prevalence of serotype 6B(4.3%) increased while that of serotype 14(13.0%) decreased [29]. The dominant prevalence serotypes of *S.pneumoniae* isolated from respiratory infection children in Shanghai study were 19F, 14, 23F, 6B and 19A, which were similar to our findings [30]. While in a Beijing study, besides serotypes 19F, 23F, and 14, the dominant prevalence serotypes included 15 and 6A serotypes as well [26]. In our study, the serotype coverage rate of PCV7, PCV10 and PCV13 were 71.9%, 71.9% and 89.9%, respectively. This finding was similar to the recent results from Shenzhen, China [31]. The increasing coverage of PCV13 was mainly for the high prevalence of serotype 19A.

Following the introduction of PCV7, serotype replacement, especially serotype 19A, has been observed worldwide [6], [9], [15]–[19]. The PCV7 has been introduced in China since 2008, but the vaccination rate is low. Despite this, serotype 19A is highly prevalent in Suzhou currently. This is likely due to selection pressure exerted through widespread antibiotics usage. In our previous study, approximately 86.0% children with respiratory infections had used one or more antibiotics before being admitted to the hospital [32]. Additionally, the large scale of internal and international migrant population may contribute substantially to the serotype replacement in Suzhou.

Pneumococcal antibiotic-resistant clones, defined by the Pneumococcal Epidemiology Network (PMEN) (http://web1.sph.emory.edu/PMEN/), have contributed to the increasing antimicrobial resistance and MDR of *S.pneumoniae* [18]. In our study, we revealed 9 clonal complexes (CCs) in total, of which CC271, CC81, CC242 and CC90 were characterized as belonging to Taiwan^19F^-14, Spain^23F^-1, Taiwan^23F^-15 and Spain^6B^-2 clones, respectively. This finding reveals an increasing trend of various international antibiotic-resistant clones co-existing in this area. Consistent with other studies from China [30], [31], [33], Taiwan^19F^-14 clones dominated with a proportion of 45.4% among all 175 pneumococcal isolates in the present study. There were as many as 8 STs in the Taiwan^19F^-14 clones, including original Taiwan^19F^-14 clone ST236 (n = 4), single locus variants ST271 (n = 36), ST1937 (n = 3), double locus variants ST320(n = 32), ST8909, ST8912, ST2648(n = 1), and triple locus variant ST9062(n = 1). Furthermore, Taiwan^19F^-14 clones were related to serogroup 19, with 33 isolates of serotype 19A and 46 isolates of serotype 19F. Importantly, ST320 with serotype 19A, the second most frequently identified clone in the current study, are high virulent and easy transmitted [34].

In the further analysis, we found that international antibiotic-resistant clones showed a significantly higher non-susceptible rate to β-lactam antibiotics than other isolates. Especially, Taiwan^19F^-14 clones had the highest non-susceptible rate to β-lactams among all the international antibiotic-resistant clones in our study. This result indicated a predominant role of Taiwan^19F^-14 clones in β-lactams resistance. In addition, 98.7% of Taiwan^19F^-14 clones were positive for both *ermB* and *mefA/E* genes, whereas only 33.3% of other clones and non-clone isolates in this study were positive for *ermB* and *mefA/E* genes. McGee et al. reported the originally Taiwan^19F^-14 clone only carries *mefA/E* gene [18].The horizontal transfer of the *ermB* gene into this clone, and subsequent dissemination of this variant, could be responsible for the high rate of the dual presence of the *ermB* and *mefA/E* genes among pneumococcal isolates in this study.

To the best of our knowledge, this is one of the first reports using multiplex PCR to detect co-colonization phenomenon from China, although it has been described elsewhere [35], [36]. Compared with serological methodology, multiplex PCR is a more sensitive, simple and cost-effective method, and it can detect the co-colonizing strains. In the present study, 3 co-colonizing strains, which contained two serotypes of 19A and 6B, were identified. However, not all the 93 pneumococcal serotypes were included in our multiplex PCR scheme. Thus, the overall co-colonization rate may be underestimated in our study.

## Conclusion

The spread of international antibiotic-resistant clones, especially Taiwan^19F^-14 clones, played a predominant role in the dissemination of antimicrobial resistant isolates in Suzhou, China. Considering the high prevalence of PCV7 serotypes and serotype 19A, the introduction of PCV13 may be a promising preventive strategy to control the increasing trend of clonal spread in China.
